# Relapse of obsessive–compulsive disorder after cerebral venous sinus thrombosis: a case report

**DOI:** 10.1007/s40211-019-00327-8

**Published:** 2019-12-11

**Authors:** Vid Velikić, Andreas Wippel, Marion Freidl

**Affiliations:** Clinical Division of Social Psychiatry, Department of Psychiatry and Psychotherapy, Medical University of Vienna, Währinger Gürtel 18–20, 1090 Vienna, Austria

**Keywords:** OCD, Cerebrovascular Insult, Sertraline, Clomipramine, Combination therapy, OCD, Zerebraler Insult, Sertralin, Clomipramin, Kombinationstherapie

## Abstract

Obsessive–compulsive disorder (OCD) is characterized by repetitive, persistent and unwanted thoughts and ritualistic, repetitive behaviors. The pathophysiology of OCD involves many distinct cortical and subcortical regions and it has been reported that OCD may occur as a consequence of traumatic brain injury, infections and tumors as well as cerebrovascular insult such as cerebral venous sinus thrombosis (CVST). We here describe the case of a 36-year-old woman who developed OCD at the age of 13 with almost complete remission of the symptoms after a 1 year-long treatment. Interestingly, after suffering CVST at the superior sagittal sinus at the age of 33, she experienced a relapse of OCD. The patient was successfully treated with Sertraline and Clomipramine. Previous studies revealed cases of OCD following different cerebrovascular accidents, i.e. predominantly arterial stroke. However, the present case is the first to describe OCD after venous thrombosis. Based on our clinical experience, the most effective treatment of OCD after CVST represents the combination of the selective serotonin reuptake inhibitor Sertraline and the tricyclic antidepressant Clomipramine.

## Zusammenfassung

Die Zwangsstörung (OCD) ist gekennzeichnet durch sich wiederholende, anhaltende und unerwünschte Gedanken sowie rituelle, sich wiederholende Verhaltensweisen. Die Pathophysiologie der OCD umfasst viele distinkte kortikale und subkortikale Regionen. Berichtet wurde, dass eine OCD als Folge von Infektionen, Tumoren, traumatischen Hirnläsionen und zerebrovaskulären Insulten, beispielweise einer zerebralen Sinusvenenthrombose (CVST), auftreten kann. Wir stellen eine 36-jährige Frau vor, die im Alter von 13 Jahren eine OCD entwickelte, wobei nach einer einjährigen Behandlung eine fast vollständige Remission der Symptome erzielt wurde. Interessanterweise erlitt sie ein Jahr nach einer CVST der V. sagittalis superior im Alter von 33 Jahren ein Rezidiv der OCD. Sie wurde mit einer Kombination aus Sertralin und Clomipramin erfolgreich behandelt. Frühere Studien zeigten Fälle von OCD nach verschiedenen zerebrovaskulären Störungen, vorwiegend nach arteriellem Schlaganfall. Die vorliegende Publikation ist jedoch die erste, in welcher eine OCD nach einer venösen Thrombose beschrieben wird. Nach unserer klinischen Erfahrung besteht die wirksamste Behandlung einer OCD nach CVST in einer Kombination des selektiven Serotoninwiederaufnahmehemmers Sertralin mit dem trizyklischen Antidepressivum Clomipramin.

## Introduction

Obsessive–compulsive disorder (OCD) is a common psychiatric disease characterized by recurrent, unwilling thoughts (obsessions) causing unpleasant feelings that drive patients to perform repetitive actions (compulsions) in order to reduce a sense of tension [1]. According to the latest studies, this disorder affects around 2–3% of the global population. Both men and women in early adulthood are equally affected. On average, however, it appears that men develop disorder symptoms earlier than women [1]. Notably, the majority of the patients with OCD respond well to pharmacotherapy and cognitive behavioral therapy (CBT) [2].

The Yale Brown Obsessive Compulsive Scale (Y-BOCS) represents one of the best validated and most commonly used diagnostic tools for accessing the severity of OCD [3], which we also used in the present study. With the maximum score of 40 points (20 for obsessions, and 20 for compulsive symptoms), patients scoring higher than 24 points are considered to suffer from a severe OCD. In general, therapy is considered effective after a symptom reduction of at least 35% according to Y‑BOCS [3].

Findings from neuroimaging studies indicate that hyperactivity in the orbitofrontal cortex (OFC) and/or dysregulation of the central serotonergic system play a role in the pathogenesis of OCD [4]. It has also been reported that OCD may occur as a consequence of pathological conditions of the central nervous system (CNS) such as traumatic brain injury, infections, and tumors. In contrast to depression and anxiety disorders, which frequently occur following a cerebrovascular accident (CVA), cases of OCD following CVA have not been frequently reported, including clinical course, treatment, and outcome. Notably, a case–control study in Sweden reported that the prevalence of OCD after suffering a stroke was 9% versus 2% in the general population, suggesting that the condition remains underdiagnosed [5].

The present study describes a female patient who initially developed OCD at the age of 13. She went almost into complete disease remission after a 1 year-long treatment. However, after suffering sinus venous thrombosis (CVST) at the age of 33, she experienced a severe relapse of OCD that was successfully treated with Sertraline and Clomipramine, in combination with CBT.

## Case presentation

Initial OCD symptoms of the patient at the age of 13 were manifested as emetophobia and obsession to be clean. Her first hospitalization at a child’s psychiatry department took place in 1996, where she was treated with paroxetine (20 mg) and thioridazine (25 mg). The treatment was successful and resulted in a long-lasting relief of the OCD symptoms. In June 2015, almost 2 decades later at the age of 33, the patient suffered acute CVST with a sudden onset of motor weakness in the left extremities and severe headache. She received acute treatment with low molecular weight heparin (LMWH) and was subsequently treated with an anticoagulant dabigatran at the department of neurology. Besides several years of using contraceptives, there were no other known risk factors for thrombosis. She was also not receiving any psychopharmacological treatment.

Three months post CVST the majority of neurological symptoms had receded and only mild hemiparesis on the left side remained. However, during the following year, the patient was, according to her description upon admission to our clinic, gradually re-developing OCD symptoms that escalated in June 2016, at the age of 34, when she was no longer able to work as a pedagogue at the day care. Her OCD symptoms were associated with extensive hands washing, showering and dressing rituals, as well as the need to control doors, locks and stove. These rituals consumed more than 8 h per day, and the symptoms were particularly intense when the patient was home alone. She even experienced difficulties to leave her house. Based on patient’s autoanamnesis and our objective assessment, there was no other plausible cause for her relapse in OCD beside the described CVST event (patient did not experience any significant change in her professional or emotional life prior to the relapse).

A thorough clinical assessment conducted at our clinic resulted in OCD diagnosis according to the International Statistical Classification of Diseases and Related Health Problems 10th Revision (ICD-10). The Y‑BOCS score was at the time 36 (20 points for obsessions and 16 points for compulsions). Notably, no clinical signs or anamnestic records were indicating potential psychiatric comorbidities. Routine blood analyses were normal. Brain magnetic resonance imaging (MRI) showed superficial siderosis and hemosiderin deposition on the right parietal lobe, which is considered to be status post hemorrhagia in the respective brain region. The patient was subsequently subjected to a combination treatment comprising Sertraline (up to 250 mg/day) and Clomipramine (up to 225 mg/day) in combination with CBT. She was closely monitored for potential adverse effects such as serotonin syndrome (e.g. restlessness, confusion, tachycardia or hypertension), and notably, no side effects occurred either during or after the treatment. Eight weeks after the therapy has been initiated, we were able to observe an initial reduction of her OCD symptoms, while one year later, the patient’s Y‑BOCS score was reduced to 16 (6 points for obsessions, and 10 points for compulsions), suggesting overall significant improvement of her OCD. Unfortunately, a complete disease remission has not been reached until the present day.

## Discussion and conclusions

In contrast to the previous studies describing correlation between cerebrovascular insult and OCD (cases of OCD following arterial stroke), our case is the first one describing a severe OCD relapse after suffering from sinus venous thrombosis [8]. CVT results in 0.5–1% of all strokes in adults with an estimated incidence of 3–4 cases per million people, while about 75% of all affected patients are women [6, 7]. CVST is an uncommon cause of cerebral infarction compared to arterial genesis, and it often remains unrecognized at the initial phase due to diversity of clinical manifestations [7]. A common MRI diagnostic finding in CVST is an abnormal signal intensity within the venous structure, which is an indication for altered flow and thrombus formation. Cases of OCD following CVA have not been frequently reported, and interestingly, it has been shown that initiation of OCD symptoms may vary from 1 month to 2 years post CVA [8]. In the present case, the patient was gradually re-developing OCD post CVST, while the maximum intensity of the symptoms was reached approximately one year later.

Recent studies have shown that combination treatment is superior to selective serotonin reuptake inhibitor (SSRI) monotherapy in treatment of OCD [9]. According to the current recommendations, treatment of OCD should be based on SSRI over an extended period of time, gradually increasing in dose up to the highest level of tolerance, or augmenting the SSRI with an agent from a different drug class [10]. The possibility of developing serotonin syndrome in a combination treatment of Clomipramine and Sertraline, as a potential complication could be highly significant for the clinical outcome, but in our case no clinical signs were observed while the patient was treated. Since our observations are based on a single case, we still cannot claim that the combination therapy used for treating our patient would be generally effective for treating OCD post CVST (in conjunction with CBT). Nevertheless, by applying the combination treatment generally recommended for OCD we were able to observe the huge benefit of the prescribed therapy, as reflected in symptom reduction measured by Y‑BOCS scale already after 12 months of treatment.

It is worth mentioning that the psychiatrists and neurologists working with patients who are suffering from CVST should be aware of the presentation of an aggravation of OCD symptoms during treatment.
